# Association between Responsible Pet Ownership and Glycemic Control in Youths with Type 1 Diabetes

**DOI:** 10.1371/journal.pone.0152332

**Published:** 2016-04-22

**Authors:** Louise Maranda, Olga T. Gupta

**Affiliations:** 1 Department of Quantitative Health Sciences, University of Massachusetts Medical School, Worcester, Massachusetts, United States of America; 2 Division of Endocrinology, Department of Pediatrics, University of Texas Southwestern Medical Center, Dallas, Texas, United States of America; Tufts University, UNITED STATES

## Abstract

Type 1 diabetes mellitus (T1DM) a chronic characterized by an absolute insulin deficiency requires conscientious patient self-management to maintain glucose control within a normal range. Family cohesion and adaptability, positive coping strategies, social support and adequate self-regulatory behavior are found to favorably influence glycemic control. Our hypothesis was that the responsible care of a companion animal is associated with these positive attributes and correlated with the successful management of a chronic illness such as type 1 diabetes. We recruited 223 youths between 9 and 19 years of age from the Pediatric Diabetes clinic at the University of Massachusetts Medical School, reviewed the status of their glycemic control (using three consecutive A1c values) and asked them questions about the presence of a pet at home, and their level of involvement with its care. Multivariate analyses show that children who care actively for one or more pets at home are 2.5 times more likely to have control over their glycemic levels than children who do not care for a pet, adjusting for duration of disease, socio-economic status, age and self-management [1.1 to 5.8], p_Wald_ = 0.032. A separate model involving the care of a petdog only yielded comparable results (OR_a_ = 2.6 [1.1 to 5.9], p_Wald_ = 0.023).

## Introduction

Type 1 diabetes mellitus (T1DM) is a chronic disease caused by an immunologic destruction of the pancreatic β-cells, resulting in an absolute insulin deficiency. It requires conscientious medical care and patient self-management education to prevent acute complications and to reduce the risk of long-term sequelae [1]. Approximately three quarters of all cases of type 1 diabetes are diagnosed in individuals younger than 18 years of age, and the American Diabetes Association (ADA) recognizes that children have characteristics and needs that dictate standards of care that are different than for adults [2].

Current standards for diabetes management reflect the need to maintain glucose control within a normal range. However, numerous reports indicate that normalization of blood glucose levels is seldom attainable in children and adolescents after the honeymoon period when insulin requirements are low [2]. Adolescence is a particularly vulnerable time for the deterioration of glycemic control due to the onset of physiologic insulin resistance coupled with the rebellious, risk-taking behavior that characterizes this time period and negatively affects self-care [2]. Diabetes self-management education, medical nutrition therapy, physical activity, psychosocial care and control of concurrent diseases are the tenets of diabetes care. In his review, Ohmann [3] names stress, inadequate social support, poor coping skills, depression, poor self-esteem and difficulty externalizing problems as factors having a negative impact on self-care and glycemic control. Conversely, family cohesion and adaptability, positive coping strategies, social support and adequate self-regulatory behavior are found to favorably influence glycemic control. One may speculate that the presence of a companion animal, capable of enhancing the positive factors named above, would augment the array of tools available for the successful management of a chronic illness such as type 1 diabetes.

Much has been written about the beneficial impact of pet ownership on human well-being. In their recent reviews, Wells [4], O’Haire [5] and McNicholas [6] categorize studies about the impact of companion animals on the basis of physical health (short and long-term effects) and psychological health. Studies about the short-term physical health effects of pet ownership point to the fact that animals serve as moderators of stress, with beneficial influences on heart rate and blood pressure. Similarly, studies about the long-term physical effects of pets seem to indicate that companion animals can 1) prevent their owners from developing illness, and 2) facilitate their owner’s recovery from ill health. In studies about the effect of companion animals on psychological health, research has shown that animals can ameliorate the effects of potentially stressful life events, reduce levels of anxiety, loneliness and depression, and enhance feelings of autonomy, competence and self-esteem [7]. Results from studies about the measurement of human-animal relationships are often tenuous, and seldom generalizable, but most support the observation that animals are beneficial additions to people’s lives [8].

In our evaluation of the published literature on health effects of human-animal relationships, less than 10% of the reviewed publications were reports about research done in children, and none were done concerning the impact of pet ownership on the control of diabetes. Specifically, the only two articles where pet ownership is studied in the context of diabetes are one measuring canine responses to hypoglycemia in adult patients with type 1 diabetes [9], and the other investigating the attitude of adult patients with type 2 diabetes toward pet ownership and dog-walking [10]. The process of caring for, loving and being loved by a companion animal could offer direct and/or indirect benefits that have yet to be specifically quantified in children with type 1 diabetes mellitus. Therefore, we investigated the relationship between responsible pet ownership and glycemic control of type 1 diabetes. We defined “responsible pet ownership” as the long-term, active control and ownership of the tasks required to maintain a happy, healthy pet, and hypothesized that it is associated with responsible management of a chronic medical condition. Our long-term goal is to uncover novel, inexpensive and feasible strategies to improve the management of youths with diabetes.

## Materials and Methods

The project described here was supported by Grant Number R03 HD071263-01 from the Eunice Kennedy Shriver National Institute of Child Health & Human Development and Mars-Waltham. This case-control study represents the first of three independent methodological constructs proposed to answer the question of interest. The second approach is a cohort study, based on incident cases of Type I diabetes, which is still ongoing. The third approach was a randomized trial called "A Novel Behavioral Intervention in Adolescents With Type 1 Diabetes Mellitus Improves Glycemic Control" (ClinicalTrials.gov Identifier: NCT01733524), published in April of 2015, in The Diabetes Educator, Vol 41:2, pp224-230. The content presented here is solely the responsibility of the authors and does not necessarily represent the official views of the *Eunice Kennedy Shriver* National Institute of Child Health and Human Development, the National Institutes of Health, or Mars-Waltham.

### Study Design and Population

Participants between the ages of 9 and 19 years were recruited for this case-control study from the Pediatric Diabetes clinic at the University of Massachusetts Medical School (UMMS, Worcester, MA) during their regular scheduled visit The standard of care at each clinic visit includes an examination by a physician or nurse practitioner and an evaluation by certified diabetes educator (CDE) to provide education tailored to the patient’s and family’s specific needs that may include the following topics: blood glucose monitoring, pattern management skills, sick day management, factors affecting blood glucose, insulin therapy, exercise, and psychosocial issues. Non-English-speaking patients and/or guardians were offered medical translation services. Participants received a $5 gift card as compensation and the entire process lasted less than 30 minutes. Signed informed consent was obtained from all participants. A parent or guardian signed the forms for children under 18; participants 18 years or older signed their own forms. Written assent was also obtained from children younger than 15years of age. The University of Massachusetts Medical School Institutional Review Board approved the study.

### Theoretical Model, Variables and Measurements

A child was classified as a *case* if s/he had achieved target glycated hemoglobin levels (HbA1c) at the three consecutive Endocrinology clinic visits immediately preceding our initial interview. Target values were based on the guidelines published in 2005 by the American Diabetes Association (ADA): Children of school age (6 to 12 years) = target A1c of 8% or less; Adolescents and young adults (between 13–19 years) = target A1c of 7.5% or less.

All other participants were classified as *controls*.

The main exposure variable was “responsible pet ownership”, measured by a questionnaire created by the research investigators (S1 Pet Ownership Questionnaire). The species and number of pet(s) owned were investigated, and the level of involvement was measured with simple questions about the type, frequency and duration of care being offered. A visual analog scale (VAS) was also offered to measure the self-perceived involvement of each child with the care of their pet(s). With this information, children were classified either as having no pet, or having a pet with which they had very little interaction, or as having a pet with which they interact fully (high involvement). Within the high involvement pet ownership group, a distinction was made between ownership of a dog and that of other animals due to the added physical activity attached to the care of a canine companion.

The Self-Management of type 1 Diabetes in Adolescent (SMOD-A) instrument [11] was also administered to all participants to measure levels of self-management of the disease. The SMOD-A assesses five dimensions: collaboration with parents, diabetes care activities, diabetes problem solving, diabetes communication and goals, each yielding an individual dimension-specific score. These scores served as adjustments for existing diabetes care status. Other potential confounding variables such as age, duration of disease and ethnic/racial characteristics were collected from the participants’ chart. Socio-economic status was established based on the mailing address, using the criteria established by Downs et al. [12]

### Statistical Analysis

Crude comparisons of the characteristics of the cases and the controls were carried out using Student’s *t* tests and/or Mann-Whitney’s U tests (Table 1 continuous variables) and Mantel-Haenszel Chi^2^ or Wald tests (Table 2 categorical variables). The relationship between the probability of having one’s diabetes under control and the quality of one’s relationship with one (or several) pet(s) was explored using multivariable statistical modeling. Logistic regression equations, linking the outcome and the exposure of interest, and adjusting for possible confounders, were specified in ascending order. Variables that when included in the model produced a change equal to or greater than 10% in the association of interest were retained as confounders. Others were removed from the final model (Table 3). Model fit was evaluated using likelihood ratio tests. All analyses were done using SPSS v. 22 (IBM, Armonk, NY).

**Table 1:** Comparison of characteristics of patients with type 1 diabetes recruited between January 2012 and April 2013, from the Pediatrics Endocrinology Clinic at the University of Massachusetts Medical School (Continuous variables).

	Controls		Cases		
Variable	(High HbA1c)		(Low HbA1c)		
	*n* = 189		*n* = 34		
	Mean ± SD	95% Confidence Interval	Mean ± SD	95% Confidence Interval	*p* value
Age (years)	15.41 ± 2.50	[15.04 to 15.77]	13.88 ± 2.34	[13.07 to 14.70]	0.001
HbA1c (%)	9.01 ± 1.19	[8.84 to 9.18]	7.14 ± 0.61	[6.93 to 7.36]	<0.001
Duration of Diabetes (years)	6.11 ± 4.18	[5.51 to 6.71]	3.81 ± 3.27	[2.67 to 4.95]	0.003
SMODA-A scores^1^					
Collaboration with Parents	21.48 ± 10.30	[20.00 to 22.97]	23.19 ± 10.30	[19.60 to 26.79]	0.376
Care Activities	32.73 ± 5.87	[31.95 to 33.62]	34.84 ± 6.28	[32.64 to 37.03]	0.076
Problem Solving	14.21 ± 3.91	[13.66 to 14.79]	13.26 ± 4.51	[11.69 to 14.83]	0.253
Communication	18.06 ± 5.44	[17.28 to 18.84]	18.74 ± 6.14	[16.59 to 20.88]	0.551
Goals	15.31 ± 3.65	[14.89 to 15.89]	15.66 ± 4.92	[13.94 to 17.37]	0.698

^1^ SMODA-A questions offer four choices between 0 and 3. Independent dimension scores will vary between 0 and 39 for “Collaboration with parents” (13 questions), between 0 and 45 for “Care activities” (15 questions), between 0 and 30 for “Communication” (10 questions) and between 0 and 21 for both “Problem solving” and “Goals” (7 questions each). Higher scores indicate more/better performance for each dimension.

**Table 2:** Comparison of characteristics of patients with type 1 diabetes recruited between January 2012 and April 2013, from the Pediatrics Endocrinology Clinic at the University of Massachusetts Medical School (Categorical variables).

	Controls	Cases		
Variable	(High HbA1c)	(Low HbA1c)	OR [95%CI]	p value
	*n* (%)	*n* (%)		(Wald)
Responsible pet ownership				
No pet or Low Level of Interaction	103 (54.5)	12 (35.3)	Reference	
High level of Interaction	86 (45.5)	22 (64.7)	2.17 [1.02 to 4.65]	0.045
Socio-Economic Status^1^				
H/A Index 1	52 (27.5)	5 (14.7)	Reference	
H/A Index 2	30 (15.9)	8 (23.5)	2.69 [0.80 to 9.07]	
H/A Index 3	33 (17.5)	8 (23.5)	2.25 [0.67 to 7.56]	
H/A Index 4	45 (23.8)	9 (26.5)	2.15 [0.67 to 6.98]	
H/A Index 5	29 (15.3)	4 (11.8)	1.36 [0.34 to 5.52]	0.510
Gender				
Females	83 (47.7)	20 (40.8)	Reference	
Males	91 (52.3)	29 (59.2)	1.75 [0.81 to 3.76]	0.153
Race and Ethnicity				
White (non-Hispanic)	154 (81.5)	34 (100.0)		
White (Hispanic)	26 (13.8)	0 (0.0)		
Non-White	9 (4.8)	0 (0.0)	Does not converge	0.002^2^

^1^ The H/A index used as a proxy for Socio-Economic status consists of 5 categories that combine the hazards/stressor exposure index (H) and the adaptive/socio-demographic character index (A) established by Downs et al. for the zip code of residence reported in patient charts.

^2^ Yate’s adjusted Chi^2^ asymptotic 2-sided.

**Table 3:** Final multivariable logistic model: estimates of simultaneous effects of active care for a pet, duration of disease, age, socio-economic status, collaboration with parents and care activities on the glycemic target status of patients (n = 223).

Variable	Coefficient	SE	Wald	Odds Ratio ^a^	95% Confidence Interval	P value (Wald)
Active care for a pet	0.914	0.427	4.583	2.49	[1.08 to 5.76]	0.032
Duration of Disease	-0.180	0.071	6.421	0.84	[0.73 to 0.96]	0.011
Age	-0.327	0.110	8.867	0.72	[0.58 to 0.89]	0.003
Socio-Economic Status			4.506			0.342
SES2	1.146	0.661	3.008	3.14	[0.86 to 11.47]	0.083
SES3	0.727	0.662	1.208	2.07	[0.57 to 7.57]	0.272
SES4	0.699	0.631	1.225	2.01	[0.58 to 6.93]	0.268
SES5	-0.087	0.759	0.013	0.92	[0.21 to 4.06]	0.909
Collaboration with parents	-0.048	0.028	2.903	0.95	[0.90 to 1.01]	0.088
Care activities	0.043	0.039	1.247	1.04	[0.97 to 1.13]	0.264
Constant	2.459	2.363	1.083	11.70		0.298

^a^ Odds ratios for one unit of each continuous variable.

## Results

Patients between the ages of 9 and 19 years with T1DM were eligible to participate which was 481 patients (73% of the total number of patients with T1DM followed in the clinic). One hundred and sixteen patients (24.12%) met our exclusion criteria which were documented mental impairment or developmental delay, severe immunological deficiency, type 2 diabetes mellitus or other comorbidities that would influence a child’s ability to care for a pet. Three hundred sixty-five eligible patients were approached to participate in our study. One hundred and forty two of these (38.9%) were not consented due to a variety of reasons: declined participation (7.7%), age greater than 19 years at the time of contact (11.8%), relocation from the clinic (8.2%) and missed clinic appointments (11.2%). Two hundred twenty-three children ages 9 to 19 years were enrolled and completed the study. One hundred and twenty of the youths (53.8%) were male, and the mean age of the patient cohort was 15.18 ± 2.53 years. Initial comparisons revealed no significant differences between the High-HbA1c and Low-HbA1c groups for gender, and socioeconomic status on the basis of health-relevant and socio-demographic heterogeneity, or scores on the SMOD-A questionnaire. The largest imbalances were “race/ethnicity”, where the Low-HbA1c group contained only non-Hispanic white patients; “age”, where the participants in the High-HbA1c group were older than the ones in the Low-HbA1c group (15.41 vs 13.88 years, p = 0.001), and “disease duration”, where the control participants had been diagnosed at an earlier age than the case participants (6.11 vs 3.81 years of disease duration, p = 0.003) (Tables 1 and 2).

Responses from our pet ownership questionnaire indicated that 43 (19.3%) respondents did not have a pet at home. Of the remaining 180 households, 84 (46.7%) had only one pet and 96 (53.3%) had two or more pets, with 26 households having multiple pets. The household with the most pets had 16 individual animals, including a goat and chickens, all considered to be pets. Dogs were the most popular pet, with 109 (60.6%) households keeping at least one (one dog = 81, two dogs = 22, three dogs = 5 and four dogs = 1), followed by cats (50.6%), rodents (rabbits, hamsters, etc.) (8.3%), fish (7.2%), reptiles (snakes, turtles, etc.) (7.2%), birds (6.7%), and amphibians (1.1%), alone or in combinations. Other pets named were horses (2), chickens (2), chinchilla (1), ferret (1), goat (1), hermit crab (1) and tarantula (1). We did not have sufficient statistical power to adjust for pet type, given that many households contained multiple combinations of pet species.

The remaining information on our questionnaire, used to assess the level of involvement of each child in the care of the pet(s) they mentioned, is not summarized here due to the large variation in responses. Of the 180 youths who had a pet at home, 70 (38.9%) were found to be minimally involved in its care. Of the remaining 110 patients who were found to have a high level of involvement with their pet, 76 reported their main pet is a dog (69.1%).

Multivariable logistic modeling (Table 3) revealed a strong adjusted relationship between HbA1c level and active pet care (OR_a_ = 2.49 [1.08 to 5.75], p_Wald_ = 0.033). Confounders were “duration of disease”, “age”, “socio-economic status”, “collaboration with parents” and “care activities”. Inclusion of the remaining variables (“gender”, “problem solving”, “communication”, “goals”) did not have an impact on the relationship of interest. No adjustment for Race/Ethnicity was possible due to lack of convergence. A separate model involving the care of a pet defined as a dog only yielded comparable results (OR_a_ = 2.59 [1.14 to 5.87], p_Wald_ = 0.023), for which none of the SMODA-A variables had any influence on the association.

## Discussion

The present study assessed the relationship between active care of a household pet and achievement of glucose control in pediatric patients with T1DM. Our findings reveal that patients with HbA1c values below the ADA target are more likely to take responsible care of a household pet than those with HbA1c values above the target.

The design of the Pet Ownership Questionnaire allowed us to query families about a variety of pets and accurately identify which aspects of the pet care were performed by the child. The majority of families were able to provide clear responses to the questions, and we were able to categorize children into distinct levels of pet care responsibility. Nearly 85% of our study participants had not reached adequate glycemic control in a consistent manner, which is typical for this pediatric patient population [13]. Similarly, we noted that the youths in the control group, who had not achieved glycemic control, were older than the participants in the case group. This is consistent with the deterioration of metabolic control that has been observed across adolescence [14] and highlights the need for efficient and cost effective tools to support this young patient population.

With our questionnaire, we were very careful to assess care, as opposed to attachment. Our hypothesis hinged on the interchangeability of caring for oneself, and caring for a pet, independently of attachment. During our interviews, we met many children who professed undying love for their pet, but were not involved in their care at all. This emphasizes our ultimate goal of providing concrete options for families affected by this chronic condition, where training and guidance can ultimately associate diabetes self-care in the child with pet maintenance and health, while no amount of instruction can control the level of attachment displayed by children toward the family pet. Of note is the fact that we were unable to adjust the relationships we uncovered for the cultural attitudes towards both pet ownership and diabetes care, since our “race/ethnicity” variable displayed complete separation. We are confident however that the complexity of our socio-economic construct adequately captures these possible effects on our relationship of interest.

Several mechanisms may be responsible for the observed relationship between active care of a household pet and achievement of glycemic control including (1) promoting an enhanced feeling of responsibility which translates to improved self-efficacy with diabetes self-care behaviors [15], (2) establishing a household routine that provides structure for habitual daily activities [16] or (3) fostering an elevated mood which has been shown to directly relate to glycemic control [17, 18]. Alternatively, the observational nature of our study may show that children who already possess a heightened sense of self may apply the same level of responsibility to the care of their pet.

In addition to the lack of directionality for our relationship, the present study faces two notable weaknesses. First, our pet ownership questionnaire has never been validated. Our goal when creating it was to capture as many elements of responsibility as possible, in an effort to objectively capture a child’s level of involvement with the care of the household pet. Our underlying assumption was that children who cannot state what their pet eats, or who never bathe or groom it, probably aren’t very involved in its care. However, the use of the visual analog scale allowed us to measure not only the child’s self-assessment of pet care, but the parent’s assessment also. High initial values were often “renegotiated” until a child-parent consensus was reached. We believe that the combination of all the elements of such a questionnaire was successful to properly classify children for our purposes, even in absence of former validation.

Second, our use of the SMODA-A only may not have provided us with a complete assessment of levels of diabetes care. Several alternative instruments are available for studying self-efficacy [19] and autonomy [20] in pediatric patients, and combining these with the SMODA-A may have enhanced our ability to control for self-care imbalances in our study population. Furthermore, other measures of mood or responsibility may have been helpful in identifying the mediators of the relationship between active pet care and glycemic control.

## Conclusion

Our data show a positive relationship between active care of a household pet and achievement of glycemic control in pediatric patients with T1DM. The identification of this relationship justifies conducting ongoing studies to identify the mechanisms mediating this association. While proposing that non-pet owning families adopt a dog or cat may be impractical, a child may benefit from responsible ownership of a fish or other low maintenance, inexpensive pet. Similarly, families that currently own a dog or cat may find that encouraging a shared responsibility of the pet with the child with diabetes could boost feelings of ownership and indirectly improve glycemic control. Associating self-care to the care of the family pet may bring about positive changes that may ultimately enhance the lives of the parents, the child and the pet.

## Supporting Information

S1 Dataset (XLSX)

S1 Pet Ownership Questionnaire (DOCX)
