# Supplementary material for: Association between Responsible Pet Ownership and Glycemic Control in Youths with Type 1 Diabetes
Source: PLoS One. 2016 Apr 22;11(4):e0152332. doi: 10.1371/journal.pone.0152332 (PMC4841520; doi:10.1371/journal.pone.0152332)
Supplement: S1 Pet Ownership Questionnaire — (DOCX) [file pone.0152332.s002.docx]

Subject ID: ___________ Date: ________________

**Pet Ownership Questionnaire**

1) Do you currently have any pet(s) in your household? □YES □NO

2) Pet species (indicate all that apply):

□Dog □Cat □Bird □Fish □Reptile (i.e. lizard, snake, turtle)

□ Amphibian (i.e. frog, newt) □ Rodent (i.e. hamster, rabbit, mouse, gerbil, rat)

□ Other ______________________________

3) Where did you acquire the pet?

□Stray □Pet store □Shelter □Breeder □Other_______________

4) Who is the owner of the pet?

□You □Parent/guardian □Sibling □Other______________

5) Who is the most responsible for the care and feeding of your pet?

□You □Parent/guardian □Sibling □Other_____________

6) Describe how you take care of your pet:

**DIET/WATER**:______________________________________________________________

**GROOMING/HYGIENE**:(i.e. baths, cleaning cage/bowl/litter box):_______________________

**EXERCISE**: ________________________________________________________________

**POTTY DUTY:** ______________________________________________________________

**OTHER**: ___________________________________________________________________

7) How much time do you spend caring for your pet(s)? __________/ day __________/ week

8) What do you normally feed your pet? ________________________________________________

9) Who buys the food for your pet? ___________________________________________________

10) What medical therapy did your pet receive at the veterinarian’s?

□Vaccine □Spay/Neuter □Treatment (i.e. flea prevention) □Other________

Visual analog Scale

| I don’t do anything for this pet | 0 **------------------------------------------------------------------------------------------------**10 | I do everything this pet needs |
| --- | --- | --- |
